# Dynamic Immune Reconstitution and Clinical Outcomes of Three Different Protocols for Haploidentical Hematopoietic Stem Cell Transplantation

**DOI:** 10.1002/mco2.70779

**Published:** 2026-05-24

**Authors:** Xiao‐Di Ma, Jie Ji, Zheng‐Li Xu, Lan‐Ping Xu, Yu Wang, Xiao‐Hui Zhang, Yu‐Qian Sun, Xiao‐Dong Mo, Yi‐Fei Cheng, Hui‐Dong Guo, Tian Dong, Xiao‐Jun Huang

**Affiliations:** ^1^ Peking University Institute of Hematology Peking University People's Hospital Peking University Beijing Key Laboratory of Hematopoietic Stem Cell Transplantation National Clinical Research Center for Hematologic Disease Beijing China; ^2^ Stem Cell Transplantation & Cellular Therapy Division Clinic Trial Center Institute of Hematology Department of Hematology West China Hospital Sichuan University Chengdu China; ^3^ Peking‐Tsinghua Center for Life Sciences Academy for Advanced Interdisciplinary Studies Peking University Beijing China

**Keywords:** Beijing protocol, conditioning regimen, haploidentical hematopoietic stem cell transplantation, immune reconstitution, posttransplant cyclophosphamide

## Abstract

The wider application of posttransplant cyclophosphamide (PTCY) and granulocyte colony‐stimulating factor (G‐CSF)/antithymocyte globulin (ATG)‐based protocols has revolutionized haploidentical hematopoietic stem cell transplantation (haplo‐HSCT) by decreasing graft‐versus‐host disease and facilitating engraftment. In this study, we compared the clinical outcomes and the immune reconstitution of propensity score‐matched (1:1:1) patients receiving PTCY (*n* = 45), ATG (*n* = 45), or PTCY plus ATG (*n* = 45). Patients in the ATG group had significantly higher overall survival (OS) (*p* = 0.029) and leukemia‐free survival (LFS) (*p* = 0.034). CD3^+^ (*p *< 0.01) and CD8+ T‐cell counts (*p* = 0.02) were greater at 3 months after transplantation in the ATG group. After adjustment for relevant covariables, Cox models revealed a significant association between CD8^+^ T‐cell reconstitution and OS in all patients (*p* = 0.008); CD8+ T‐cell recovery and LFS showed a similar trend (*p* = 0.034). Sensitivity analysis revealed stable results. Restricted cubic spline curve analysis to visualize the relationship between immune reconstitution and outcomes revealed that the CD8^+^ T‐cell count at 3 months post‐HSCT strongly correlated with survival prognosis. These findings demonstrate that conditioning regimens profoundly impact immune reconstitution, which may contribute to differences in survival prognosis. Moreover, increasing the probability of CD8^+^ T‐cell reconstitution after HSCT may become an important strategy for improving outcomes.

## Graphical abstract

This study compared clinical outcomes and immune reconstitution in haplo‐HSCT patients receiving PTCY (*n* = 45), ATG (*n* = 45), or PTCY+ATG (*n* = 45). The ATG group had superior OS (*p* = 0.029) and LFS (*p* = 0.034), with higher CD3+ and CD8+ T‐cell counts at 3 months. We suggested that CD8+ T‐cell recovery significantly correlated with survival, highlighting its potential as a therapeutic target.

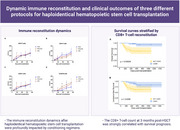

## 1 Introduction

In recent years, haploidentical hematopoietic stem cell transplantation (haplo‐HSCT) has emerged as a promising therapeutic option for various hematologic malignancies, with significant global expansion driven by the advent of innovative techniques to address the challenge of human leukocyte antigen (HLA) disparity—a primary contributor to delayed immune reconstitution, graft rejection, and severe graft‐versus‐host disease (GvHD) [1]. Advances in haplo‐HSCT methodologies, including various T‐cell depletion techniques, such as high‐dose posttransplant cyclophosphamide (PTCY) administration [2] and G‐CSF/antithymocyte globulin (ATG)‐based protocols [3], have led to clinical outcomes comparable to those of HLA‐matched donor transplantation [4, 5, 6, 7]. However, the immunomodulatory mechanisms of these platforms are different. While ATG‐based and PTCY‐based regimens both aim to facilitate engraftment, they affect donor T‐cell dynamics through different biological pathways, leading to distinct patterns and kinetics of immune reconstitution. Furthermore, combined PTCY–ATG strategies have been investigated to better balance immunological tolerance and recovery. Understanding how these specific platforms influence immune recovery is therefore essential to linking prophylaxis choices with clinical outcomes.

Immune reconstitution is a critical factor for long‐term outcomes after HSCT, which influences the risk of relapse, infection, and GvHD [8, 9, 10, 11]. Numerous factors can impact immune reconstitution, including age, sex, histocompatibility, stem cell source, conditioning regimen, and GvHD prophylaxis strategies [12, 13, 14]. Typically, immune recovery follows a staged timeline: innate immune cells recover within weeks to months after transplantation, whereas adaptive immune cells occur more slowly. While B cells and CD8^+^ T cells generally reappear within the first 100 days to 6 months, the restoration of thymus‐dependent CD4^+^ T cells is a much more protracted process. Importantly, conditioning regimens and GvHD prophylaxis strategies can deeply affect specific immune compartments, resulting in distinct patterns and kinetics of immune recovery.

According to current evidence, the recovery of specific lymphocyte subsets, rather than total lymphocyte counts, is critical for the success of transplantation. In several transplant settings, early reconstitution of cytotoxic CD3^+^CD8^+^ T cells has been associated with improved survival [10, 15], suggesting that CD8^+^ T cells serve as predictors of survival. Other studies have emphasized the significance of CD4^+^ helper T‐cell recovery [16, 17]. Meanwhile, they suggested that the relative contributions of CD8^+^ and CD4^+^ T cells may be context‐dependent and influenced by the graft source, conditioning intensity, and HLA disparity.

However, direct comparisons of immune reconstitution across different haplo‐HSCT platforms remain limited [18, 19]. More importantly, immune reconstitution may serve as a biological bridge linking conditioning and GvHD prophylaxis strategies to long‐term clinical outcomes; however, this mechanistic relationship has not been fully elucidated. In many previous studies, immune recovery has been analyzed using arbitrary dichotomization of continuous variables, an approach that may obscure nonlinear associations and fail to capture the dynamic complexity of immune recovery. Therefore, in this study, we aimed to compare immune reconstitution kinetics across different haplo‐HSCT protocols and to identify critical immune cell subsets that may mediate differences in prognosis while controlling for baseline characteristics via multigroup propensity score matching (PSM). In addition, we used Cox regression analysis, sensitivity analysis, and restricted cubic splines (RCSs) as highly effective and visually intuitive approaches to explore the relationship between immune reconstitution and clinical outcomes. Our method may provide a more sophisticated understanding of posttransplant immune recovery biology, which may lead to more prompt therapies and better monitoring techniques for improved patient outcomes.

## 2 Results

### 2.1 Patient Characteristics

A total of 135 patients were included in this retrospective study: 45 in the PTCY group, 45 in the PTCYATG group, and 45 in the ATG group. The baseline characteristics of the patients in the three groups are shown in Table 1. Except for the MNC and CD34+ cell counts, the characteristics were comparable among the three groups after PSM. The median follow‐up times in the PTCY, PTCYATG, and ATG groups were 458 days (range, 10–1276), 456 days (range, 28–800), and 611 days (range, 68–1474), respectively.

**TABLE 1:** Patient, disease, and transplant characteristics.

Characteristic	Total (*N* = 135)	PTCY (*N* = 45)	PTCYATG (*N* = 45)	ATG (*N* = 45)	*p*
Patient age, y (median, range)	38 (3, 61)	34 (15, 60)	42 (14, 61)	34 (3, 65)	0.159
Patient sex, *n* (%)					0.915
Male	63 (46.7%)	21 (46.7%)	20 (44.4%)	22 (46.7%)	
Female	72 (53.3%)	24 (53.3%)	25 (55.6%)	23 (53.3%)	
Disease type, *n* (%)					0.861
ALL	42 (31.1%)	15 (33.3%)	16 (35.6%)	11 (24.4%)	
AML	62 (45.9%)	22 (48.9%)	19 (42.2%)	21 (46.7%)	
MDS	21 (15.6%)	5 (11.1%)	7 (15.6%)	9 (20.0%)	
Others	10 (7.4%)	3 (6.7%)	3 (6.7%)	4 (8.9%)	
HCT‐CI					0.683
0–1	121 (89.6%)	42 (93.3%)	40 (88.9%)	39 (86.7%)	
≥ 2	14 (10.4%)	3 (6.7%)	5 (11.1%)	6 (13.3%)	
Disease status before HSCT					0.752
CR1	70 (51.9%)	22 (48.9%)	25 (55.6%)	23 (51.1%)	
CR2/CR3	33 (24.4%)	12 (26.7%)	8 (17.8%)	13 (28.9%)	
PR/NR	32 (23.7%)	11 (24.4%)	12 (26.7%)	9 (20.0%)	
Disease risk index					0.366
Low risk	1 (0.7%)	0	0	1 (2.2%)	
Intermediate/high risk	128 (94.8%)	41 (91.1%)	44 (97.8%)	43 (95.6%)	
Very high risk	6 (4.4%)	4 (8.9%)	1 (2.2%)	1 (2.2%)	
Conditioning regimen					0.871
MAC	129 (95.6%	43 (95.6%)	44 (97.8%)	42 (93.3%)	
RIC	6 (4.4%)	2 (4.4%)	1 (2.2%)	3 (6.7%)	
Donor‐recipient sex match, *n* (%)					0.401
Male to male	38 (28.1%)	11 (24.4%)	10 (22.2%)	17 (37.8%)	
Female to female	28 (20.7%)	11 (24.4%)	11 (24.4%)	6 (13.4%)	
Female to male	24 (17.8%)	9 (20.0%)	10 (22.2%)	5 (11.1%)	
Male to female	45 (33.3%)	14 (31.1%)	14 (31.1%)	17 (37.8%)	
ABO match, *n* (%)					0.709
Matched	76 (56.3%)	26 (57.8%)	29 (64.4%)	21 (46.7%)	
Major mismatched	31 (23.0%)	9 (20.0%)	9 (20.0%)	13 (28.9%)	
Minor mismatched	21 (15.6%)	7 (15.6%)	6 (13.3%)	8 (17.8%)	
Different	7 (5.2%)	3 (6.7%)	1 (2.2%)	3 (6.7%)	
Number of HLA‐A/B/DRB1 mismatches, *n* (%)					0.305
1	14 (10.4%)	3 (6.7%)	4 (6.7%)	8 (17.8%)	
2	54 (40.0%)	16 (35.6%)	21 (46.7%)	17 (37.8%)	
3	67 (49.6%)	26 (57.8%)	21 (46.7%)	20 (44.4%)	
MNC, ×10^8^/kg, M(range)	11.25 (3.58, 34.63)	11.71 (3.58, 34.63)	14.46 (6.00, 26.87)	10.92 (6.79, 32.03)	0.001
CD34, ×10^8^/kg, M(range)	5.47 (0.52, 17.30)	6.05 (2.4, 17.7)	5.90 (0.52, 17.30)	5.51 (0.92, 13.32)	0.001

Abbreviations: ALL, acute lymphoblastic leukemia; AML, acute myeloid leukemia; CR, complete remission; HLA, human leukocyte antigen; MAC, myeloablative conditioning; MDS, myelodysplastic syndrome; MNC, mononuclear cell; NR, non‐remission; PR, partial remission; RIC, reduced‐intensity conditioning.

### 2.2 Clinical Outcomes

**FIGURE 1:**
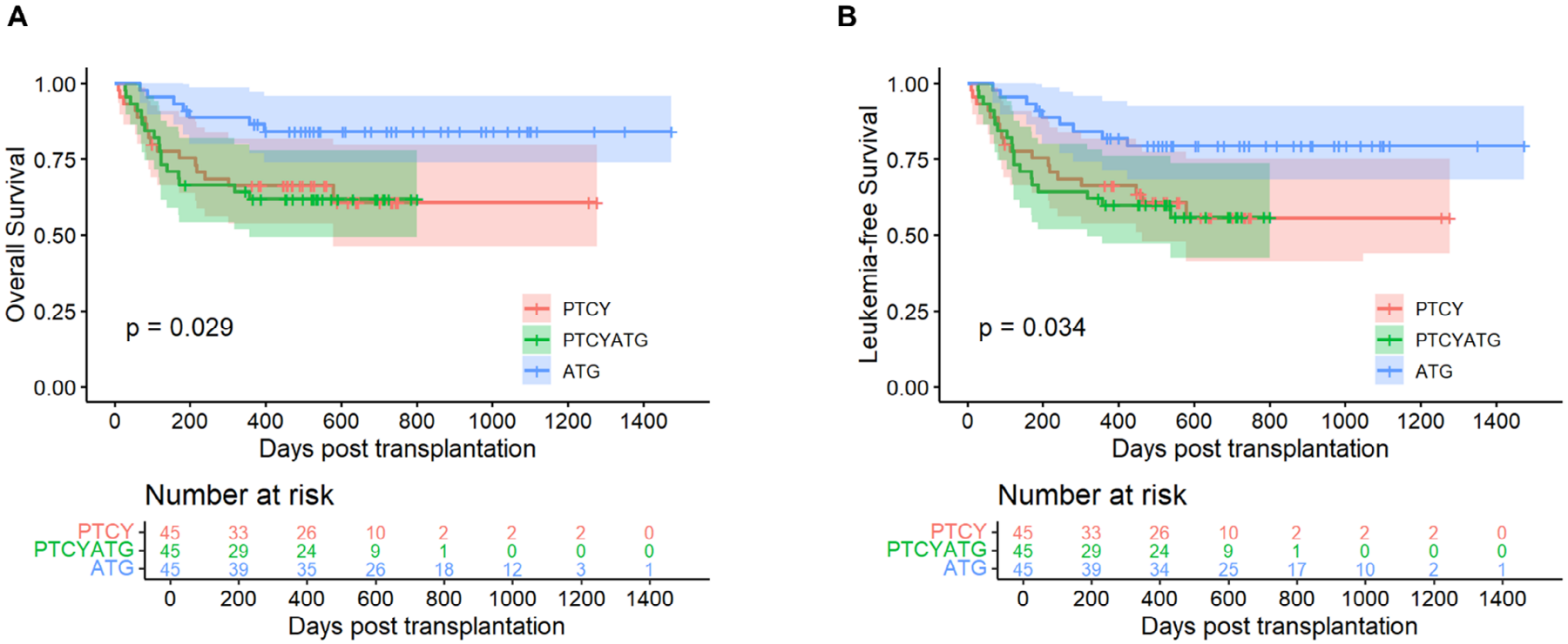
The 3‐year (A) overall survival (OS) and (B) leukemia‐free survival (LFS) of patients after haplo‐HSCT with three different conditioning regimens.

The posttransplant clinical outcomes of the three groups are summarized in Table 2 and Figure 1. At the end of the follow‐up period, the OS probability was significantly higher in the ATG group (84.2%, 95% CI: 74.0%–95.7%) than in the PTCY group (60.8%, 95% CI: 46.4%–79.6%) or the PTCYATC group (62.0%, 95% CI: 49.2%–78.0%) (*p* = 0.029; Figure 1A). Similarly, compared with the other two protocols, the ATG approach was associated with a significantly higher LFS rate (*p* < 0.01). The cumulative incidence of LFS was 55.8% (95% CI: 41.5%–74.9%) in the PTCY group, 55.9% (95% CI: 42.5%–73.6%) in the PTCYATG group, and 79.5% (95% CI: 68.4%–92.4%) in the ATG group (Figure 1B). The 100‐day cumulative incidence rates of Grades II–IV aGvHD were 37.8% (95% CI: 25.4%–53.5%) in the PTCY group, 40.0% (95% CI: 27.4%–55.7%) in the PTCYATG group, and 31.1% (95% CI: 19.8%–46.8%) in the ATG group (*p* = 0.604). The cumulative incidence of Grade III–IV aGvHD at 100 days was also comparable across the treatment strategies (17.8% for PTCY, 26.7% for PTCYATG, and 22.2% for ATG; *p* = 0.628). A total of 2, 3, and 4 patients in the PTCY, PTCYATG, and ATG groups, respectively, experienced relapse after HSCT. The three approaches yielded comparable relapse rates: 5.4% (95% CI: 1.4%–20.1%) for PTCY, 12.3% (95% CI: 4.6%–30.6%) for PTCYATG, and 11.2% (95% CI: 4.8%–24.9%) for ATG (*p* = 0.562). The 3‐year cumulative incidence of NRM was 38.7% (95% CI: 25.0%–56.4%) in the PTCY group, 33.3% (95% CI: 21.6%–49.1%) in the PTCYATC group, and 11.2% (95% CI: 4.8%–24.9%) in the ATG group (*p* = 0.034).

**TABLE 2:** Cumulative incidences (95% CI) of clinical outcomes in the three groups.

Clinical outcomes	PTCY	PTCYATG	ATG	*p*
CMV infection	80.0 (67.4%–90.1%)	77.7 (64.9%–88.5%)	55.6 (41.8%–70.3%)	0.061
EBV infection	2.2 (0.3%–14.7%)	17.8 (9.3%–32.4%)	15.6 (7.7%–29.9%)	0.049
3‐year relapse	5.4 (1.4%–20.1%)	12.3 (4.6%–30.6%)	11.2 (4.8%–24.9%)	0.562
3‐year NRM	38.7 (25.0%–56.4%)	33.3 (21.6%–49.1%)	11.2 (4.8%–24.9%)	0.012
100‐day aGVHD				
II–IV aGVHD	37.8 (25.4%–53.5%)	40.0 (27.4%–55.7%)	31.1 (19.8%–46.8%)	0.604
III–IV aGVHD	17.8 (9.3%–32.4%)	26.7 (16.1%–42.2%)	22.2 (12.6%–37.4%)	0.628
3‐year OS	60.8 (46.4%–79.6%)	62.0 (49.2%–78.0%)	84.2 (74.0%–95.7%)	0.029
3‐year LFS	55.8 (41.5%–74.9%)	55.9 (42.5%–73.6%)	79.5 (68.4%–92.4%)	0.034

Abbreviations: aGVHD, acute graft‐versus‐host disease; CI, confidence interval; CMV, cytomegalovirus; EBV, Epstein–Barr virus; LFS, leukemia‐free survival; NRM, non‐relapse mortality; OS, overall survival.

### 2.3 Dynamic Immune Reconstitution

**FIGURE 2:**
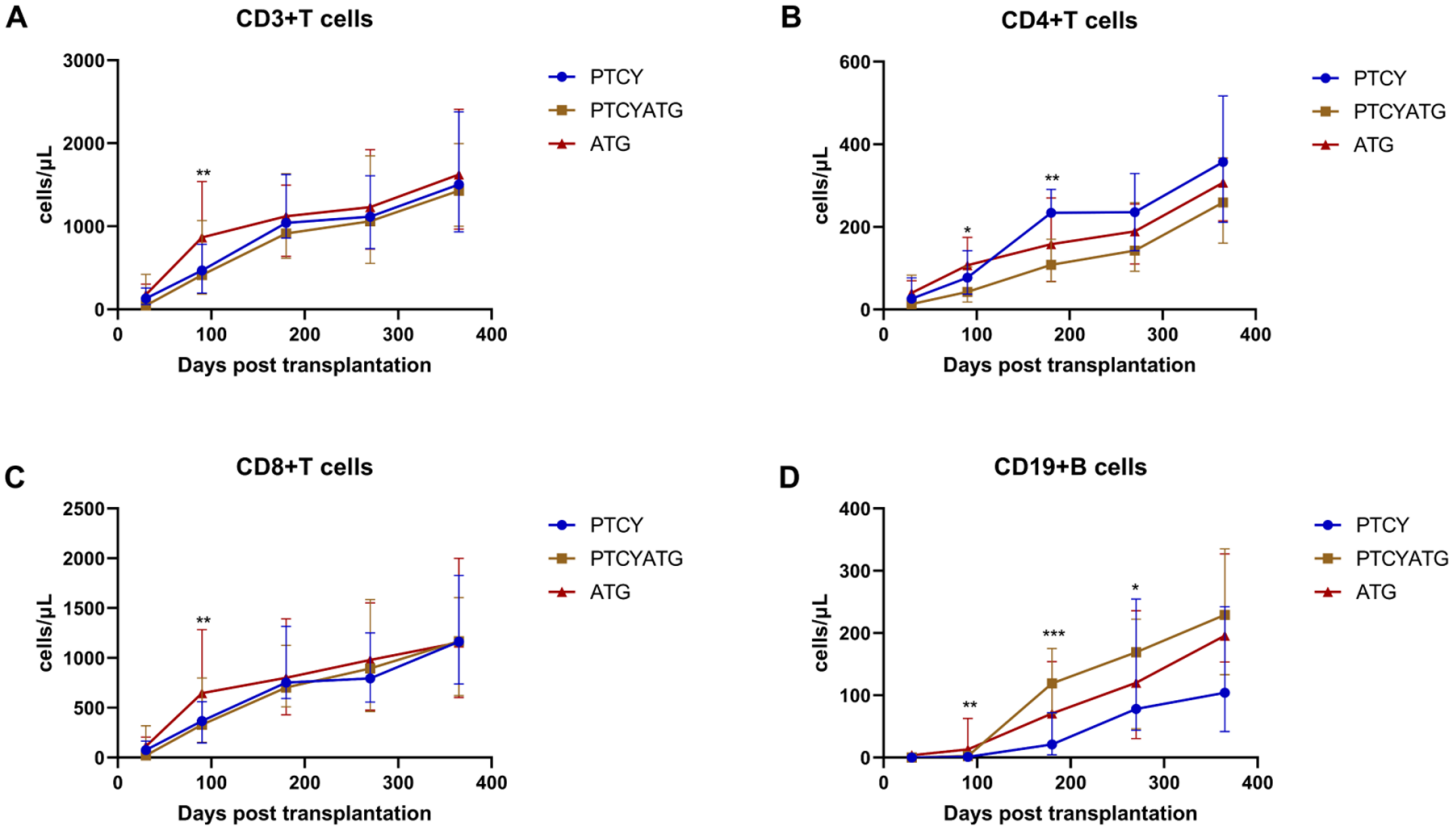
Immune reconstitution kinetics: Median counts of four immune cell subsets in patients after HSCT with three protocols at 1, 3, 6, 9, and 12 months after transplantation. The error bars indicate the 25th–75th percentiles. (A) CD3^+^ T cells; (B) CD4^+^ T cells; (C) CD8^+^ T cells; and (D) CD19^+^ B cells. *p < 0.05, **p < 0.01, ***p < 0.001.

In this study, we measured the counts of CD3^+^ T, CD4^+^ T, CD8^+^ T, and CD19^+^ B cells at 1, 3, 6, 9, and 12 months post‐haplo‐HSCT. The immune recovery dynamics and absolute values of immune cell subsets at each time point are presented in Figure 2 and Table 3.

**TABLE 3:** Immune cell subset counts of the three groups after transplantation (median [25th–75th]).

Time after HSCT (months)	CD3^+^T cells (cell/µL)				CD4^+^T cells (cell/µL)			
	PTCY	PTCYATG	ATG	*p*	PTCY	PTCYATG	ATG	*p*
1	130.50 (58.50–257.00)	46.50 (16.80–420.50)	176.37 (113.65–303.37)	0.18	26.00 (15.25–76.75)	13.00 (2.00–83.30)	40.48 (21.13–69.77)	0.23
3	469.00 (195.50–784.00)	412.50 (183.80–1067.80)	866.98 (361.01–1539.10)	<0.01	77.00 (37.00–142.00)	42.50 (18.00–103.00)	107.44 (49.74–174.61)	0.02
6	1040.00 (858.50–1624.00)	913.00 (617.00–1634.00)	1121.20 (638.18–1495.34)	0.68	234.00 (185.50–290.75)	108.00 (67.00–170.00)	158.40 (68.37–270.45)	0.01
9	1116.50 (730.25–1607.50)	1061.50 (553.75–1061.50)	1232.02 (727.25–1923.54)	0.85	235.50 (142.25–329.25)	142.50 (92.80–258.80)	189.12 (110.43–255.36)	0.10
12	1503.84 (934.00–2379.00)	1429.50 (1000.50–1995.80)	1623.06 (966.14–2407.63)	0.94	357.00 (211.00–517.00)	259.00 (160.80–367.30)	307.32 (214.85–366.68)	0.32

Time after HSCT (months)	CD8^+^T cells (cell/µL)				CD19^+^B cells (cell/µL)			
	PTCY	PTCYATG	ATG	*p*	PTCY	PTCYATG	ATG	*p*
1	76.50 (44.50–166.00)	22.50 (12.50–319.50)	112.63 (55.12–206.20)	0.28	0.00 (0.00–0.25)	0.00 (0.00–0.75)	3.72 (1.42–6.84)	<0.01
3	367.00 (146.00–561.00)	331.00 (152.80–799.50)	646.08 (327.24–1284.59)	0.02	1.00 (1.00–3.00)	2.50 (0.25–9.80)	13.05 (4.22–62.98)	<0.01
6	752.50 (592.00–1316.75)	703.00 (510.00–1127.00)	800.04 (430.95–1392.98)	0.89	21.00 (4.50–72.00)	119.00 (70.50–175.30)	70.87 (19.01–154.28)	0.03
9	795.50 (557.25–1251.75)	894.00 (461.80–1586.00)	979.74 (475.14–1553.56)	0.96	78.00 (43.75–254.50)	169.00 (46.00–222.30)	120.21 (30.38–235.93)	0.87
12	1163.00 (739.00–1829.00)	1168.50 (621.50–1605.00)	1155.88 (603.20–1998.33)	0.98	104.00 (41.75–242.25)	229.00 (133.00–335.00)	195.69 (153.65–327.02)	0.07

Overall, the major differences in immune reconstitution among the three protocols were concentrated within the first 3 months after transplantation. At 1 month posttransplantation, the PTCYATG group presented the lowest absolute number of CD3^+^ T cells (Figure 2A), with a median count of 46.50, whereas the CD3^+^ T‐cell count was comparable between the PTCY (130.50) and ATG groups (176.37). However, by the third month after HSCT, compared with the other two groups, the ATG group (866.98) presented significantly higher numbers of CD3^+^ T cells (469.00 in the PTCY group and 412.50 in the PTCYATG group) (*p* < 0.01). Beyond this time point, the CD3^+^ T‐cell counts in the PTCY and PTCYATG groups recovered quickly and were comparable to those in the ATG group at 6, 9, and 12 months post‐HSCT. A similar pattern was observed for CD8+ T cells (Figure 2C). At 1 month posttransplantation, the CD8^+^ T‐cell counts remained low across the three groups. However, the ATG group demonstrated a significantly faster recovery rate by the third month (646.08 in the ATG group, 367.00 in the PTCY group, and 331.00 in the PTCYATG group) (*p* < 0.01). After this point in time, there were no statistically significant differences in cell counts among the three groups.

The absolute counts of CD4^+^ T cells in all three groups were significantly lower than those of CD8^+^ T cells during the first month posttransplantation (26.00 in the PTCY group, 13.00 in the PTCYATG group, and 40.48 in the ATG group). By the third month posttransplantation, the CD4^+^ T‐cell count was higher in the ATG group than in the other two groups (107.44 in the ATG group, 77.00 in the PTCY group, and 42.50 in the PTCYATG group; *p* = 0.02). However, the PTCY group experienced rapid expansion of CD4+ T cells, which reached a count of 234.00 by 6 months posttransplantation; this T‐cell count was significantly higher than that observed in the ATG group (158.40) and in the PTCYATG group (108.00) (*p* = 0.01; Figure 2B).

Among all the cell types analyzed here, CD19^+^ B cells recovered the slowest after HSCT, nearly disappearing initially but then experiencing rapid expansion by 6 months after haplo‐HSCT (Figure 2D). At 3 months posttransplantation, the absolute B‐cell counts were higher in the ATG group than in the other two groups (13.05 in the ATG group, 1.00 in the PTCY group, and 2.50 in the PTCYATG group; *p* < 0.01). At 6 months post‐HSCT, the absolute number of CD19^+^ B cells (119) was highest in the PTCYATG group.

Considering the overall trends in immune reconstitution summarized above, all three groups showed a gradual restoration of immune cell subsets starting from the third month posttransplantation. Notably, the ATG group demonstrated superior early reconstitution at 3 months, whereas the PTCY group showed relatively rapid recovery of CD4+ T cells beginning at 6 months.

### 2.4 Impact of Immune Reconstitution on Survival Outcomes

Univariate Cox regression analysis was conducted to explore the associations between immune cell subsets at different stages (as continuous variables) and OS or LFS (Supporting Information, Table S1). The results revealed that T‐cell reconstitution at the third month post‐HSCT, particularly CD8^+^ T‐cell reconstitution, was significantly associated with OS (*p* = 0.004) and LFS (*p* = 0.015).

Weighted Cox regression models were subsequently constructed to verify the correlation between CD8^+^ T‐cell reconstitution and survival outcomes among patients who received HSCT, and the results are presented in Table 4. Model 2 was adjusted for age, sex, and diagnosis, and Model 3 was adjusted for the same variables as Model 2, plus HCT‐CI and disease status. When CD8^+^ T‐cell reconstitution was analyzed as a continuous variable, the HRs and 95% CIs of OS in Models 1, 2, and 3 were 0.165 (0.046–0.594), 0.156 (0.043–0.561), and 0.185 (0.053–0.642), respectively, all of which demonstrated a significant negative correlation with the OS (all *p* < 0.05). Similarly, the HRs and 95% CIs for LFS in Models 1, 2, and 3 were 0.409 (0.197–0.846), 0.395 (0.185–0.844), and 0.436 (0.202–0.940), respectively.

**TABLE 4:** Associations between immune reconstitution of CD8^+^ T cells at 3 months posttransplantation and survival outcomes (OS, LFS).

	CD8^+^T cells at 3‐month posttransplantation							*p* for trend
	Continuous^d^		T1	T2		T3		
Overall survival (OS)								
Model 1^a^	0.165 (0.046, 0.594)	0.006	1 (reference)	0.169 (0.048, 0.602)	0.006	0.120 (0.027, 0.536)	0.006	0.017
Model 2^b^	0.156 (0.043, 0.561)	0.004	1 (reference)	0.163 (0.046, 0.584)	0.005	0.108 (0.023, 0.497)	0.004	0.013
Model 3^c^	0.185 (0.053, 0.642)	0.008	1 (reference)	0.195 (0.053, 0.713)	0.013	0.100 (0.018, 0.553)	0.008	0.019
Leukemia‐free survival (LFS)								
Model 1^a^	0.409 (0.197, 0.846)	0.016	1 (reference)	0.198 (0.064, 0.610)	0.005	0.193 (0.063, 0.597)	0.011	0.051
Model 2^b^	0.395 (0.185, 0.844)	0.016	1 (reference)	0.193 (0.063, 0.597)	0.004	0.259 (0.089, 0.755)	0.013	0.061
Model 3^c^	0.436 (0.202, 0.940)	0.034	1 (reference)	0.222 (0.071, 0.697)	0.010	0.293 (0.089, 0.960)	0.043	0.091

*Note*: The results are presented as HRs and 95% CIs.

Abbreviations: CI, confidence interval; HR, hazard ratio; T, tertile.

^a^ Model 1: crude.

^b^ Model 2: further adjusted for age, sex, and diagnosis.

^c^ Model 3: adjusted for the same variables as in Model 2 and further for HCT‐CI (hematopoietic cell transplantation‐specific comorbidity index) and disease status.

^d^ The cell counts were standardized using the *z* score before being included in the Cox regression analysis; the hazard ratio represents a 1‐SD increase (standard deviation) in the cell count.

In the sensitivity analyses, the CD8^+^ T‐cell count was categorized into tertiles (T1, T2, and T3). In the unadjusted Model 1 for OS, the HRs (95% CIs) for T2 and T3 compared with T1 were 0.169 (0.048–0.602) and 0.120 (0.027–0.536), respectively; both tertiles were significantly associated with OS. After adjustments for age, sex, and diagnosis in Model 2, the HRs (95% CIs) for T2 and T3 compared with T1 were 0.163 (0.046–0.584) and 0.108 (0.023–0.497), respectively, with significant differences observed for T2 and T3 compared with T1. In the fully adjusted Model 3, the HRs (95% CIs) for T2 and T3 were 1.39 (0.91–2.13) and 1.88 (1.23–2.87), respectively, with a significant association persisting for both T2 and T3. Analyses across the tertiles for all three models revealed statistically significant trends (*p* < 0.05).

With respect to LFS, the HRs (95% CIs) for T2 and T3 were significantly different from those for T1 for all three models (all *p* < 0.05). However, the trend analysis did not reveal a statistically significant difference. To explore this relationship, we conducted an RCS analysis to gain deeper insights into the association between CD8+ T‐cell reconstitution and survival prognosis.

### 2.5 RCS Analysis

**FIGURE 3:**
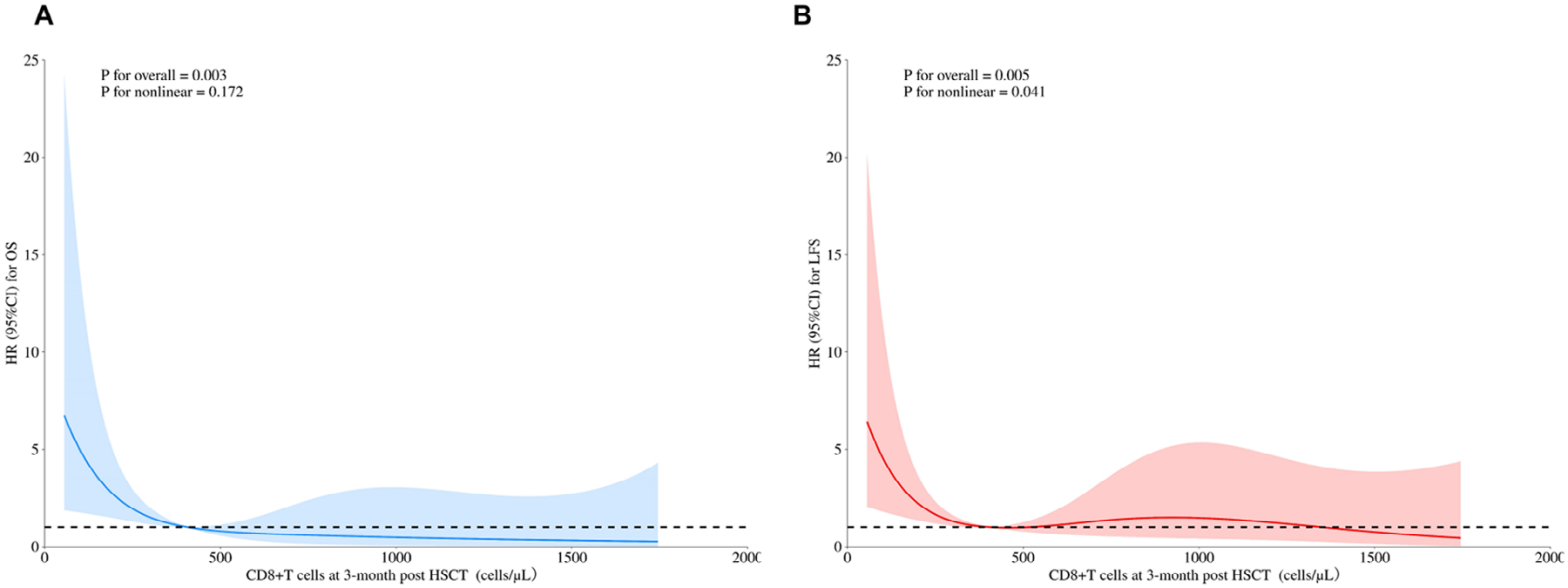
RCS curve fit between CD8^+^ T cells at 3 months posttransplantation and survival outcomes. The solid lines represent smooth curve fits between variables. Shaded bands represent 95% confidence intervals from the fit. (A) CD8^+^ T cells and OS; (B) CD8^+^ T cells and LFS.

RCS analysis revealed a strong correlation between the CD8^+^ T‐cell count at 3 months and survival. There was a linear relationship between CD8^+^ T‐cell reconstitution and OS (*p* for overall trend = 0.003; *p* for nonlinearity = 0.172; Figure 3A) and a nonlinear relationship between CD8^+^ T‐cell reconstitution and LFS (*p* for overall trend = 0.005; *p* for nonlinearity = 0.041; reflection point 407; Figure 3B).

**FIGURE 4:**
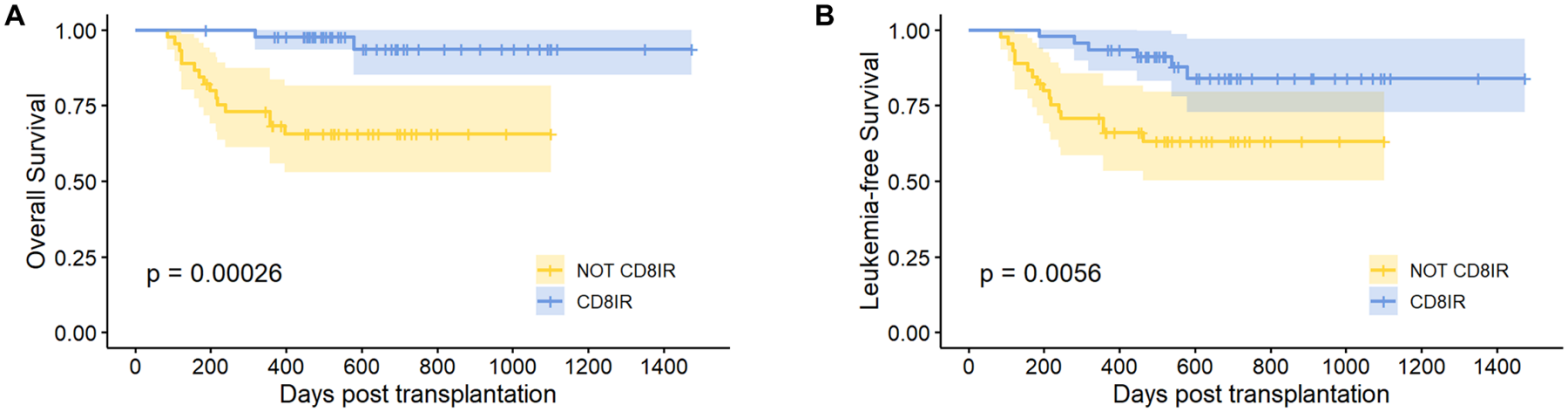
The 3‐year (A) overall survival and (B) leukemia‐free survival in the CD8‐IR group and NOT CD8‐IR group.

Overall, as the number of cells increased, the HR for survival gradually decreased. In particular, when the CD8^+^ T‐cell count increased from 0 to 407, the HR of LFS rapidly decreased; however, as the cell count continued to increase, this trend became more gradual. These findings suggested that a CD8^+^ T‐cell count of 407 might be the threshold for survival outcomes. Patients were divided into two groups on the basis of a 3‐month CD8^+^ T‐cell threshold of 407; patients with counts of 407 or higher were characterized as CD8‐IR, and those with counts below this threshold were characterized as NOT CD8‐IR. Kaplan–Meier curve analysis revealed that the OS and LFS of patients in the CD8‐IR group were significantly longer than those of patients in the NOT CD8‐IR group (log‐rank *p* < 0.01; Figure 4A, B).

## 3 Discussion

The wider application of the PTCY and Beijing protocols has revolutionized haplo‐HSCT by decreasing the incidence of GvHD and facilitating engraftment [1, 20, 21], leading to survival rates similar to or better than those of stem cell transplants from siblings, matched unrelated donors, or cord blood (CB) [4, 5, 22, 23]. Several studies, including ours, have compared the clinical outcomes of patients receiving different treatment regimens [24, 25]. However, data concerning the impact of each protocol on immune reconstitution remain scarce [19, 26]. In this two‐center, retrospective study, we compared the clinical outcomes and immune recovery kinetics after haplo‐HSCT with three different protocols. Most importantly, we explored the impact of immune reconstitution on survival outcomes after transplantation in an RCS model. The findings of this study suggest that patients undergoing haplo‐HSCT should be continuously monitored at various time points to identify those at high risk of death or relapse.

In the present study, monitoring immune reconstitution at different time points revealed the recovery trends of various immune cell subsets following different conditioning protocols. CD3^+^ T cells and CD8^+^ T cells demonstrated a notable reconstitution advantage starting at 3 months posttransplantation in the ATG group, whereas the PTCY group exhibited the fastest recovery of CD4^+^ T cells beginning at 6 months posttransplantation. Chang et al. reported that the median CD8^+^ T‐cell counts after haplo‐HSCT with the Beijing protocol reached 98, 672, 918, and 884 cells/µL at 30, 90, 180 days, and 1 year posttransplant, respectively [27]. For haplo‐HSCT using the PTCY protocol, Raiola et al. reported CD8^+^ T‐cell recovery to a median count of 107 cells/µL by Day 100 [28]. Retière et al. compared the patterns of immune recovery between the PTCY and ATG protocols and reported that the CD8+ T‐cell count remained low in the PTCY group but reached the normal range (300–700 cells/µL) at 2 months post‐HSCT in the ATG group [18]. Similarly, Massoud et al. reported that CD8^+^ T cells recovered more quickly than CD4^+^ T cells did, with evident CD8^+^ reconstitution by Day 100 but incomplete CD4^+^ T‐cell recovery on Day 180. PTCY appeared to spare CD4^+^ T cells while targeting CD8^+^ T cells more aggressively, thereby enabling CD4^+^ T‐cell proliferation. In contrast, ATG has a stronger effect on memory CD8^+^ and CD4^+^ T cells, resulting in immune reconstitution patterns distinct from those of PTCY [19]. Servais et al. examined the impact of ATG on immune reconstitution after allogeneic HSCT and reported that ATG depleted naive CD4^+^ cells, which is consistent with our results [29]. These findings suggest that PTCY has a weaker effect on CD4^+^ T cells but a stronger effect on CD8^+^ T cells, as evidenced by the higher numbers of CD4^+^ T cells in the PTCY group and CD8^+^ T cells in the ATG group.

The reconstitution of T cells following allogeneic HSCT involves two key mechanisms: homeostatic peripheral expansion (HPE) of donor‐derived T cells and thymic generation of new naive T cells [30]. HPE predominantly favors CD8^+^ T cells over CD4^+^ T cells because of the superior proliferative capacity of CD8^+^ T cells. An earlier multi‐omics study by our group involving single‐cell RNA sequencing revealed that CD8^+^ T cells in haplo‐SCT recipients are predominantly reconstituted through the central pathway, facilitating immune homeostasis, whereas reconstitution in matched sibling recipients relies more on the peripheral pathway, potentially limiting T‐cell receptor (TCR) diversity and increasing relapse risk [31]. They also reported that ATG promoted the expansion of CD8^+^ Trp cells and depleted CD8^+^ Teff cells to some extent, but the bias in T‐cell reconstitution pathways was influenced primarily by HLA disparity [31].

Tsai et al. reported that patients who underwent transplantation with the G‐CSF/ATG protocol had significantly improved OS compared with those who received PTCY plus ATG or PTCY alone (48.9% vs. 38.1% vs. 22.0%) [24]. Our findings are consistent with those of this prior study: after controlling for the baseline characteristics, the ATG group exhibited better OS and LFS and lower NRM than the other two groups did. Notably, in our cohort, the PTCYATG group had the lowest early T‐cell recovery, particularly at 1 month, and this disadvantage persisted at 3 months for both CD3+ and CD8+T cells. These findings suggest that the combined immunosuppressive intensity of PTCY plus ATG may have delayed early immune reconstitution, thereby offsetting its potential clinical benefit. Moreover, after the entire cohort was stratified on the basis of the median CD8^+^ T‐cell count at 3 months posttransplant, the OS and LFS significantly differed between the CD8‐IR and NOT CD8‐IR groups. Similarly, Tian et al. reported that the recovery of CD8^+^ cytotoxic T cells on Day +90 to a cutoff value of 375 cells/µL correlated with bacterial infections, NRM, LFS, and OS [10]. We speculate that the superior survival outcomes in the ATG group might be associated with the advantage of this protocol in T‐cell reconstitution at 3 months, potentially reducing the risk of bacterial and viral infections or relapse. However, more precise hypotheses require validation through well‐designed prospective studies.

Several studies have confirmed the role of CD8^+^ T cells in protecting recipients from infections and leukemia relapse [15, 32]. In our cohort, early CD8+ T‐cell recovery at 3 months was also associated with improved survival outcomes. Biologically, early reconstitution of CD8^+^ cytotoxic T cells may enhance host defense against bacterial and viral pathogens through interferon‐γ production and cytolytic activity, thereby reducing infection‐related non‐relapse mortality (NRM) [33]. In addition, reconstituted CD8^+^ T cells may contribute to graft‐versus‐leukemia (GVL) effects during the early posttransplant period, when immune surveillance is critical. These mechanisms provide a plausible biological explanation linking early CD8^+^ T‐cell recovery to the improved survival outcomes observed in our cohort. Accordingly, improving the probability of CD8+ reconstitution after transplantation may become an important strategy for improving the outcomes of HSCT. In a multicenter randomized controlled trial involving 220 patients, Gao et al. demonstrated that recombinant human G‐CSF combined with a minimal dose of decitabine significantly reduced the risk of AML recurrence. In addition, they reported an increase in the numbers of NK cells and CD8^+^ T cells following G‐CSF therapy, indicating enhanced GVL capacity [34].

This study has several limitations. First, this was a retrospective study that included data from only two centers. Moreover, monitoring immune reconstitution is not a standard process after HSCT, which may have introduced information bias to this study. Finally, detailed phenotypic and functional characterization of immune cell subsets, including T helper cell subsets (Th1, Th2, and Th17), regulatory T cells, and dendritic cell populations, was not systematically performed and therefore could not be performed in the present cohort. However, the current PSM method enabled close matching of baseline data for each group, and RCS analysis enabled a more logical correlation analysis between immune reconstitution and survival. An ongoing prospective clinical study in which comprehensive immune profiling has been integrated into the study design has been initiated. This approach includes longitudinal monitoring of functional T‐cell subsets and antigen‐presenting cell populations, which will enable a more detailed characterization of immune reconstitution dynamics.

In conclusion, different protocols for haplo‐HSCT have strong differences in terms of outcomes and immune reconstitution dynamics. The G‐CSF/ATG protocol appears to offer advantages in terms of OS and LFS for Chinese patients with hematologic malignancies, and this benefit appears to be driven by the faster reconstitution of CD8^+^ T cells at 3 months post‐HSCT. These findings provide valuable insights into the differences in immune reconstitution dynamics following haplo‐HSCT. Careful monitoring of immune cell subsets is critical for detecting potential post‐HSCT problems and choosing the best time for intervention.

## 4 Methods

### 4.1 Patient Selection

Patients with hematologic malignancies who underwent their first haplo‐HSCT at Peking University People's Hospital (PUKPH) or West China Hospital between January 2020 and December 2022 were included in the study. This group of patients was from a cohort that was previously reported in the Chinese Bone Marrow Transplantation Registry Group (CBMTRG) study [25]. Because immune monitoring is not part of the standard post‐HSCT process, only patients with regular post‐HSCT immune assessments were included in the present analysis: 51 in the PTCY group, 75 in the PTCYATG group, and 572 in the ATG group. To balance the baseline data, a multigroup PSM method was applied [35] with the variables of age at HSCT, patient sex and hematopoietic cell transplantation‐comorbidity index (HCT‐CI), resulting in a final cohort structure of 45:45:45 in the PTCY, PTCYATG and ATG groups.

### 4.2 Definitions

The primary endpoint of this study was leukemia‐free survival (LFS), which was defined as the time from transplantation to either death or relapse, whichever occurred first. The secondary endpoints included engraftment, acute GvHD (aGvHD), cytomegalovirus (CMV) viremia, Epstein–Barr virus (EBV) viremia, relapse, NRM, and overall survival (OS).

Neutrophil engraftment was defined as the first of three consecutive days on which the neutrophil count reached or exceeded 0.5 × 10^9^/L posttransplantation. Platelet engraftment was defined as the first of seven continuous days on which the platelet count, without transfusion, reached 20 × 10^9^/L. aGvHD was defined and graded on the basis of the modified Seattle–Glucksberg criteria, whereas chronic GvHD (cGvHD) was assessed according to the National Institutes of Health (NIH) guidelines. CMV viremia was diagnosed when CMV DNA levels in peripheral blood exceeded 5 × 10^2^/L, and EBV viremia was diagnosed when EBV DNA levels surpassed 1 × 10^3^/L; both CMV and EBV DNA levels were measured by real‐time quantitative PCR. Relapse was defined as having ≥ 5% bone marrow (BM) blasts or the reappearance of extramedullary leukemia after complete remission (CR) was achieved. NRM was defined as death resulting from causes other than relapse or disease progression. OS was defined as the interval from transplantation to death or the last follow‐up.

### 4.3 Transplantation Procedures

Transplantation was performed as described previously [25].

(1) Patients in the G‐CSF/ATG group received cytarabine (4 g/m^2^/day, Days −10 to −9), busulfan (3.2 mg/kg/day, Days −8 to −6), cyclophosphamide (1.8 g/m^2^/day, Days −5 to −4), Me‐CCNU (250 mg/m^2^ orally, Day −3), and ATG (2.5 mg/kg/day, Days −5 to −2); and GvHD prophylaxis included cyclosporine A (CsA), mycophenolate mofetil (MMF), and short‐term methotrexate (MTX).

(2) Patients in the PTCY group were given 30–50 mg/kg/day PTCY on Days +3 and +4, and the conditioning regimens included busulfan (3.2 mg/kg/day, Days −6 to −3), fludarabine (30 mg/m^2^/day, Days −6 to −2), and cytarabine (1 g/m^2^/day, Days −6 to −2) or busulfan (130 mg/m^2^/day, Day −7), fludarabine (30 mg/m^2^/day, 6 days), and melphalan (100 mg/m^2^/day, Day −2). GvHD prophylaxis included PTCY, CsA, MTX, and MMF.

(3) Patients in the PTCYATG group received high‐dose PTCY (30–50 mg/kg/day) and low‐dose rabbit ATG (1–2.5 mg/kg, Days −2, −1, or +8). The conditioning regimen consisted of busulfan (3.2 mg/kg/day, Days −6 to −3), fludarabine, and cytarabine or an alternative regimen with busulfan (130 mg/m^2^/day, Day −7), fludarabine, and melphalan (100 mg/m^2^/day, Day −2). GvHD prophylaxis included CsA, MMF, PTCY, and ATG.

### 4.4 Immunophenotype Analysis

Peripheral blood samples were obtained from patients before transplantation and at 1, 3, 6, 9, and 12 months posttransplant. Flow cytometric immunophenotyping was prospectively conducted to assess immune recovery. Directly conjugated monoclonal antibodies—CD3‐FITC, CD4‐PE, CD8‐APC, and CD19‐Per‐CP (BD Biosciences, Mountain View, CA, USA)—along with corresponding isotype controls were used to assess the immunophenotypes of the T lymphocyte subsets. Flow cytometry analysis was conducted on a BD FACS machine (Becton Dickinson Biosciences, San Jose, CA, USA), and data processing was performed using CellQuest software (BD Biosciences). T cells were identified on the basis of CD3 positivity, with subsets defined as CD3^+^CD4^+^ and CD3^+^CD8^+^. B cells were characterized as CD3^−^, CD14^−^, CD11b^−^, and CD19^+^ cells. Immune cell subsets were characterized using flow cytometry by following previously published methods [27].

### 4.5 Statistical Analysis

Categorical variables were analyzed using either the chi‐square test or Fisher's exact test, whereas nonparametric methods (the Mann–Whitney test for two groups and the Kruskal–Wallis test for comparisons among more than two groups) were employed for continuous variables. The immune cell counts and proportions are presented as medians with interquartile ranges (25th–75th percentiles). Immune cell counts at various time points were compared between groups using the Kruskal–Wallis test.

Survival outcomes were estimated with the Kaplan–Meier method and compared with the log‐rank test. The cumulative incidence of engraftment, GvHD, relapse, and NRM was calculated considering competing risks, and the Fine–Gray test was employed to assess significant differences.

To explore the correlation between immune reconstitution and survival outcomes, we constructed three weighted Cox regression models. No covariates were adjusted for in Model 1; Model 2 was adjusted for age, sex and diagnosis; and Model 3 was adjusted for age, sex, diagnosis, disease status, and the HCT‐CI. CD8^+^ T‐cell counts at 3 months posttransplantation were analyzed primarily using tertile‐based categorization to enhance clinical interpretability and, in parallel, as a continuous variable (per 1‐standard deviation increase). Sensitivity analyses were conducted by comparing the results across continuous and tertile‐based Cox models. In addition, RCS modeling was applied to explore potential nonlinear associations, with four prespecified knots placed at the 5th, 35th, 65th, and 95th percentiles of the CD8^+^ T‐cell distribution.

Statistical significance was determined using two‐sided tests with a *p*‐value threshold of < 0.05. Estimates with 95% confidence intervals (CIs) are provided. Analyses were conducted using SPSS 26.0, GraphPad Prism 8.0.1, and R (version 4.4.1) (http://www.r‐project.org).

## Author Contributions

X.‐J.H. designed the study. X.‐D.M., J.J., and Z.‐L.X. led the data collection, analysis, and primary manuscript development. L.‐P.X., Y.W., X.‐H.Z., Y.‐Q.S., X.‐D.M., Y.‐F.C., H.‐D.G., and T.D. provided the patient data. All the authors read and approved the final manuscript.

## Funding

This work was supported by the Major Program of the National Natural Science Foundation of China (No. 82293630), the National Key Research and Development Program of China (No. 2022YFA1103300), the Key Program of the National Natural Science Foundation of China (No. 82530009), the Peking University Medicine Fund for the world's leading discipline or discipline cluster development (No.71003Y3035), the National Natural Science Foundation of China (No. 82100227).

## Ethics Statement

This study was approved by the institutional review board of PKUPH and West China Hospital (approval number: 2022PHB236‐001).

## Consent

Informed consent was obtained from the patients or their families.

## Conflicts of Interest

The authors declare no conflicts of interest.

## Supporting information




**Table S1**: Univariable analysis of immune reconstitution with clinical outcomes.
**Table S2**: Univariable analysis of factors associated with clinical outcomes.
**Table S3**: Death causes in total cohort and each group.

## Data Availability

The data that support the findings of this study are available upon reasonable request from the corresponding author.
