# Supplementary material for: Dynamic Immune Reconstitution and Clinical Outcomes of Three Different Protocols for Haploidentical Hematopoietic Stem Cell Transplantation
Source: MedComm (2020). 2026 May 24;7(6):e70779. doi: 10.1002/mco2.70779 (PMC13239405; doi:10.1002/mco2.70779)
Supplement: Supplementary file 1 — Table S1: Univariable analysis of immune reconstitution with clinical outcomes. Table S2: Univariable analysis of factors associated with clinical outcomes. Table S3: Death causes in total cohort and each group. [file MCO2-7-e70779-s001.docx]

Table S1. Univariable analysis of immune reconstitution with clinical outcomes.

| **Outcomes** | | **Overall survival** | **Leukemia-free survival** |
| --- | --- | --- | --- |
| **+1m** | CD3 | .481 | .375 |
|  | CD4 | .824 | .935 |
|  | CD8 | .672 | .458 |
|  | CD19 | **.000** | **.002** |
| **+3m** | CD3 | **.006** | **.019** |
|  | CD4 | .251 | .209 |
|  | CD8 | **.004** | **.015** |
|  | CD19 | **.026** | .113 |
| **+6m** | CD3 | .966 | .659 |
|  | CD4 | .913 | .537 |
|  | CD8 | .870 | .662 |
|  | CD19 | .133 | .259 |
| **+9m** | CD3 | .160 | .145 |
|  | CD4 | .094 | .038 |
|  | CD8 | .217 | .213 |
|  | CD19 | .209 | .126 |
| **+12m** | CD3 | .293 | .650 |
|  | CD4 | .083 | .088 |
|  | CD8 | .443 | .293 |
|  | CD19 | .267 | .084 |

Table S2. Univariable analysis of factors associated with clinical outcomes.

| **Characteristics** | **Grade II-IV aGvHD** | | **Grade III-IV aGvHD** | | **Relapse** | | **NRM** | | **OS** | | **LFS** | |
| --- | --- | --- | --- | --- | --- | --- | --- | --- | --- | --- | --- | --- |
|  | HR (95%CI) | P | HR (95%CI) | P | HR (95%CI) | P | HR (95%CI) | P | HR (95%CI) | P | HR (95%CI) | P |
| Sex (male vs female) | 0.80 (0.52-1.25) | .335 | 1.26 (0.67-2.36) | .474 | 0.88 (0.36-2.12) | .773 | 1.14 (0.64-2.02) | .653 | 1.14 (0.67-1.93) | .632 | 1.02 (0.62-1.66) | .943 |
| Age (＜50 vs ≥50) | 0.99 (060-1.64) | .967 | 0.76 (0.39-1.51) | .436 | 0.32 (0.13-0.78) | .**012** | 0.44 (0.25-0.77) | **.004** | 0.41 (0.24-0.67) | **.001** | 0.40 (0.25-0.65) | **.000** |
| Disease status (CR1 vs others) | 0.95 (0.78-1.17) | .632 | 0.90 (0.67-1.21) | .479 | 1.03 (0.66-1.60) | .901 | 0.76 (0.58-0.99) | **.040** | 0.75 (0.59-0.96) | **.023** | 0.79 (0.63-1.00) | **.045** |
| DRI  (low/intermediate vs high/very high) | 0.85 (0.70-1.04) | .122 | 0.78 (0.58-1.04) | .089 | 0.93 (0.60-1.45) | .753 | 0.51 (0.38-0.68) | **.000** | 0.55 (0.42-0.71) | **.000** | 0.60 (0.47-0.76) | **.000** |
| HCT-CI (0 *vs*.≥1) | 1.27 (0.81-1.99) | .307 | 0.95 (0.51-1.77) | .870 | 1.06 (0.43-2.61) | .909 | 2.14 (1.11-4.15) | **.024** | 1.92 (1.06-3.47) | .**031** | 1.61 (0.94-2.73) | .081 |
| HLA mismatch (3 vs 1/2) | 0.85 (0.57-1.28) | .443 | 0.96 (0.54-1.73) | .899 | 1.00 (0.43-2.36) | .996 | 1.02 (0.61-1.73) | .935 | 1.07 (0.66-1.74) | .796 | 1.03 (0.65-1.62) | .908 |
| Donor-recipient sex match  (Female-Male vs Others) | 0.98 (0.64-1.50) | .939 | 0.98 (0.54-1.81) | .960 | 1.04 (0.42-2.57) | .937 | 0.94 (0.55-1.63) | .837 | 1.03 (0.61-1.72) | .919 | 0.91 (0.57-1.47) | .707 |
| Conditioning regimen |  |  |  |  |  |  |  |  |  |  |  |  |
| ATG | 1 |  | 1 |  | 1 |  | 1 |  |  |  |  |  |
| PTCY | 1.35 (0.81-2.26) | .250 | 1.16 (0.51-2.66) | .720 | 0.61 (0.18-2.07) | .430 | 4.37 (2.16-8.84) | **.000** | 2.85 (1.54-5.27) | **.001** | 2.57 (1.45-4.55) | **.001** |
| PTCYATG | 1.26 (0.78-2.01) | .350 | 1.91 (1.01-3.62) | **.047** | 0.58 (0.19-1.76) | .340 | 3.63 (1.84-7.19) | **.000** | 2.37 (1.33-4.23) | **.003** | 2.07 (1.20-3.54) | **.008** |
| IR at 90 days (25^th^) |  |  |  |  |  |  |  |  |  |  |  |  |
| CD3 (≥358.288 vs＜358.288) | 0.82 (0.46-1.47) | .512 | 0.44 (0.20-0.95) | **.037** | 0.89 (0.29-2.73) | .834 | 0.22 (0.10-0.48) | **.000** | 0.26 (0.13-0.52) | **.000** | 0.32 (0.17-0.60) | .**000** |
| CD4 (≥41.466 vs＜41.466) | 0.70 (0.40-1.25) | .227 | 0.43 (0.20-0.94) | **.034** | 1.43 (0.41-4.99) | .571 | 0.34 (0.15-0.74) | **.007** | 0.49 (0.24-1.01) | .054 | 0.56 (0.29-1.07) | .078 |
| CD8 (≥257.529 vs＜257.529) | 0.81 (0.45-1.45) | .472 | 0.37 (0.17-0.79) | **.010** | 1.28 (0.37-4.46) | .699 | 0.22 (0.10-0.47) | **.000** | 0.26 (0.13-0.52) | .**000** | 0.36 (0.19-0.67) | .**001** |
| CD19 (≥1 vs＜1) | 1.06 (0.51-2.17) | .883 | 1.31 (0.43-3.98) | .633 | 0.42 (0.15-1.20) | .105 | 0.49 (0.18-1.36) | .173 | 0.51 (0.21-1.22) | .132 | 0.46 (0.21-0.99) | **.046** |

CMV, cytomegalovirus; EBV, Epstein-Barr virus; aGVHD, acute graft-versus-host disease; NRM, non-relapse mortality; LFS, leukemia-free survival; and OS, overall survival.

Table S3 Death causes in total cohort and each group.

| Death causes | Number of cases in total cohort, percent | PTCy group | PTCy-ATG group | ATG group | *P* |
| --- | --- | --- | --- | --- | --- |
| Infection | 29 (72.5%) | 14 | 13 | 2 | **0.003** |
| Graft failure | 1 (2.5%) | 1 | 0 | 0 | 0.999 |
| GVHD | 5 (12.5%) | 1 | 1 | 3 | 0.618 |
| Organ failure | 4 (10.0%) | 0 | 2 | 2 | 0.546 |
| Others/unknown | 1 (2.5%) | 0 | 1 | 0 | 0.999 |
| Total | 40 | 16 | 17 | 7 |  |

Abbreviations: GVHD, acute graft-versus-host disease.
